# Photodynamic effect of Zirconium phosphate biocompatible nano-bilayers containing methylene blue on cancer and normal cells

**DOI:** 10.1038/s41598-019-51359-7

**Published:** 2019-10-17

**Authors:** Reza Hosseinzadeh, Khatereh Khorsandi

**Affiliations:** 1 Department of Medical Laser, Medical Laser Research Center, Yara Institute, ACECR, Tehran, Iran; 2 Department of Photodynamic, Medical Laser Research Center, Yara Institute, ACECR, Tehran, Iran

**Keywords:** Drug delivery, Nanotechnology in cancer

## Abstract

Pharmaceutical applications of methylene blue, especially as photosensitizer, have been limited due to its rapid enzymatic reduction in the biological systems. In this study nano-platelet zirconium phosphate was synthesized and its biocompatibility was evaluated. The synthesized material was considered as drug delivery vehicle for methylene blue to enhance the photodynamic therapy efficacy in human breast cancer cells. Zirconium phosphate-methylene blue nano-hybrids were characterized by X-Ray Powder Diffraction (XRPD), Scanning Electron Microscopy (SEM), and Thermo gravimetric Analysis (TGA). Biocompatibility of synthesized nano materials were studied on Hu02 human fibroblast normal cell and MDA-MB-231 human breast cancer cell. The results clarified that ZrP-MB nanoparticles could decrease the dark toxicity of free methylene blue. Photodynamic therapy using zirconium phosphate-methylene blue on MDA-MB-231 human breast cancer was evaluated by MTT assay, colony forming ability assay, AO/EB dual staining and flow cytometry detection of apoptosis. The results suggest that zirconium phosphate-methylene blue nano-hybrids significantly enhance photodynamic therapy efficacy probably via apoptosis cell death mechanism against human breast cancer cells. According to the results, zirconium phosphate nanoparticles could be suggested as a promising nano-carrier for photosensitizer delivery in photodynamic therapy.

## Introduction

Cancer is still one of the most challenging diseases and is the major cause of death after cardiovascular diseases. Breast cancer is one of the most common malignancies in women and second lethal cancer after lung cancer among females^1–4^. With development of knowledge about cancer diseases, many advances have been made to treat it^2,3,5^. Chemotherapy is one of major therapy methods and remains as common important tools in the metastatic breast cancer treatment^6–13^. Toxic effects of anticancer drugs on normal cells are still an important issue because they often act nonspecific^14–16^. Over the past two decades, new drug delivery systems have been developed that can overcome chemotherapy problems^17–20^. In recent years, more attention has been paid to the provision of nanoparticles as carriers for drug delivery. Nanoparticle carriers improve the function of drugs and reduce their side effects by changing the pharmacokinetic properties^21,22^. For synthesis of nanoparticles, various materials such as polymers, metal particles, lipids, etc. could be used which produce different shapes and sizes of nanoparticles. These structures have ability to control the release of drug, protection of drug molecule, particle size smaller than cells, cross biological barriers for reaching to the target site, increasing drug shelf life in the bloodstream, targeted drug delivery and biocompatibility which could be consider as highly effective delivery systems to increasing therapeutic efficacy of the drugs^23–26^. Recently, the use of inorganic nanomaterials for biomedical applications has been grown compared to traditional polymeric drug delivery systems. Among the variety of nanoparticles, inorganic layered compounds have a special interest in researches due to their high ion exchange capacity, crystalline structure, tunable particle size, non-spherical shapes and biocompatibility^27–30^.

Non-spherical inorganic layered structured nanomaterials (LSNs) are used in various research. There are many scientific reports on the applications of LSNs^31^. Among them, the zirconium phosphate layered cation exchange materials have been widely used in biomedical applications as drug vehicles because of their harmlessness to humans and not to interfere with the metabolic function of the body^32^. The size of synthesized ZrP materials can be easily controlled by synthesis and modification methods. They should show better binding properties, cell adhesion and margination than spherical nanoparticles due to platelet-like shape of zirconium phosphate nano-structures^33^. ZrP can intercalate wide variety of drugs and due to ion exchange ability; it can release the interacted drugs in the acidic conditions such as cancer cells microenvironment^34–36^. The lysosome and the peroxisomes could dissociate zirconium phosphate to phosphate ions and harmless zirconium salts^34^. In addition, good biocompatibility, bioactivity, cheap availability, simple synthesis method and high stability of zirconium phosphate provides the potential for their use in biomedical. Nowadays, these materials are very attractive nano-carriers for delivery of drugs, proteins, and genes^32,34^.

Photodynamic therapy (PDT) is a non-invasive radiation therapy which use for treatment of types of cancers, cardiovascular and ophthalmic diseases. PDT is based on photo activation of light sensitive material (photosensitizer). The activated photosensitizers transfer their excess energy to the surrounding oxygen to form the reactive oxygen species (ROS), particularly singlet oxygen as cytotoxic reactive materials, which will cause damage in cancer cells and tissues^4,37–39^. Another great advantage of PDT is its potential on overcoming multidrug resistance (MDR) due to specific cytotoxicity mechanism of photosensitizers onto cancer cells which is different from chemotherapy agent’s mechanisms^40–42^. The significant side effects of photosensitizer are nonspecific interactions and damage to normal cells and tissues due to low selectivity to specific cells, environmental photosensitizer degradation and water insolubility or molecular hydrophobicity which cause various limitations in clinical application of photodynamic therapy^4,39^. Application of nanoparticles in photosensitizer delivery to tumor cells in PDT has been improved. Advantages such as stable aqueous dispersion of hydrophobic photosensitizers, surface modification ability, targeted therapy, protecting photosensitizers from environmental degradation and quenching can be achieved by using nano-delivery systems^43^. According to the chemical and redox properties of methylene blue (MB), the clinical use of MB has been hindered due to simple reduction of MB to photodynamic inactive reduced form “leuco-methylene blue” in biological systems. The two major reducing systems accompanied with inactivating of methylene blue are the presence of a transmembrane thiazine dye reductase at the cell surface and intercellular reduction by NADH/NADPH dehydrogenases^44^. In this study, we have synthesized and characterized zirconium phosphate layered nano-sheets and used them as methylene blue nano-carriers for photodynamic therapy of human breast cancer cells. The loaded amounts of MB were determined by spectrophotometric and thermal methods and *in vitro* release of the drug from nano-delivery system was evaluated. *In vitro* cytotoxicity and death mechanism of ZrP-MB nanoparticles on photodynamic treatment of MDA-MB-231 human breast cancer cell line was determined.

## Materials and Methods

Zirconyl chloride (ZrOCl_2_, 8H_2_O), phosphoric acid and Methylene blue were obtained from Merck. DMEM medium (Dulbecco’s Modified Eagle Medium) was purchased from Invitrogen. MTT assay reagent, (3-(4,5-dimethylthiazol-2-yl)-2,5-diphenyltetrazolium bromide), was supplied by Sigma-Aldrich. Trypan blue solution (0.4% w/v) and dimethyl sulfoxide (DMSO) were achieved from Merck Company. Fetal bovine serum (FBS) and antibiotics were purchased from Gibco (Gibco BRL). All chemicals were of analytical grade. Double distilled deionized water was used for all experiments and solutions. All pH measurements were made at 25 °C using Bel PHS3-BW (BEL ENGINEERING, Italy). The UV-Vis absorption spectra were recorded using Cary 60 UV/Vis spectrophotometer, equipped with quartz cells. Red light emitting LED (660 nm; power density: 30 mW cm^−2^) was used as light source for photodynamic experiments.

### Synthesis of zirconium phosphate

Synthesis of Zirconium phosphate was done based on procedures reported previously in literatures^45,46^. Briefly, a certain amount of Zirconyl chloride octahydrate was dissolved in deionized water (14 mM) and stirred vigorously until complete dissolution. The resulting homogeneous solution was added dropwise to a diluted phosphoric acid solution (28 mM) and vigorously stirred. The pasty precipitate was produced. The resulting mixture was transferred to the sealed teflon-lined stainless steel autoclave. The autoclave was kept at 180–200 °C for 9 h and then cool down to room temperature under ambient conditions. The synthesized products were collected by centrifugation. For removing other contaminants from final product, the collected precipitate was re-suspended in deionized water and collected by centrifugation for three times.

### Characterization of synthesized zirconium phosphate

Characterization of the synthesized materials was done using several analytical methods. the crystal structure of ZrP was characterized using X-Ray powder diffraction analysis. XRD patterns were obtained by a Rigaku MiniFlex X-ray diffractometer using CuKα radiation (λ = 0.154 nm). The divergence, receiver, and detector slits width were 2 mm; the scatter slit width was 0.6 mm. SEM images were obtained using scanning electron microscope for powder samples by Zeiss SEM instrument (Zeiss EVO-18, Germany). EVO 18 offers Energy and Wavelength Dispersive Spectroscopy (EDS & WDS) for surface elemental analysis. The Specific Surface Area (SSA) obtained and calculated for synthesized nanoparticles (65.8 m^2^/g). Fluorescence spectra was recorded by Cary Eclipse fluorescence spectrophotometer equipped with a thermostatically controlled cell holder at ambient temperature. The excitation and emission slits were set at 5 nm. Cary 100 UV-vis spectrophotometer, equipped with quartz cuvettes was used for recording UV-Vis absorption spectra.

### Methylene blue intercalation in layered zirconium phosphate

Methylene blue was intercalated into zirconium phosphate layers according to previous methods^32^. The certain amount of zirconium phosphate suspended in the solutions contains constant concentrations of methylene blue. The experimental suspensions are kept in dark and shacked continuously. The absorbance of centrifuged samples supernatant was measured in 30 minutes’ time intervals until reaching to constant value as indicative of the end of methylene blue intercalation. The optimum time concerned with intercalation process obtained by continuous measurements of methylene blue absorbance in the supernatants at various times to obtaining intercalation equilibrium time. After reaching the intercalation equilibrium, the suspension was centrifuged and supernatant discarded. The obtained precipitate washed three times for removing excess free MB (un-intercalated MB) from intercalation materials.

### Thermo gravimetric analysis (TGA)

Thermal gravity analysis was recorded using STA 1500, from Rheometic Scientific Co. The percentage of loaded methylene blue into ZrP nanoparticles was calculated by thermo gravimetric analysis. The thermal weight losses were recorded by increasing of temperature up to 600 °C by the ramp of 5 °C/min.

### Cellular experiments

Human normal fibroblast cells (HU02) and human breast cancer cells (MDA-MB-231) were purchased from the Institute of Pasture, Tehran, Iran. DMEM medium supplemented with 10% FBS, 100 IU/ml penicillin, and 100 mg/ml of streptomycin was used as cell culture medium. The cells were grown in the supplied medium and then incubated in a humidified incubator containing 5% CO_2_ at 37 °C. The cells were removed by trypsinizing (trypsin 0.025%, EDTA 0.02%) and washed with phosphate buffer solution and then cultured for experimental purposes. For treatments, the MB and ZrP-MB solutions prepared freshly using PBS buffer solution.

### MTT assay

Colorimetric MTT (Thiazolyl blue tetrazolium bromide) assay was used for cell viability evaluation. Living cells convert the MTT to an insoluble formazan. The resulting formazan solubilized using dimethyl sulfoxide (DMSO) and its concentration measured using ELISA reader. Briefly, culture medium was removed and cells were incubated in medium containing 0.5 mg/mL of 3-(4,5-Dimethylthiazol-2-yl)-2,5-Diphenyltetrazolium bromide for 4 h at 37 °C. The resulting purple formazan crystals dissolved in 100 µL DMSO and shacked for 15 min. The absorbance of solutions was measured at 570 nm by an ELISA reader (Hyperion, Inc., FL, USA).

### Photodynamic experiments

The MDA-MB-231 human breast cancer cells were grown in medium culture and after reaching 80∼90% confluence the cells were washed with PBS, afterwards detached from the flask by addition of 1.0 mL of 0.25% trypsin for 1–3 min at 37 °C. Cells (1 × 10^4^ cells/well) were seeded into 96-well plates. The cells were treated with analyts at different conditions. After a further incubation 1 h, one plate was considered as dark (no irradiation/control plate) and the other plates illuminated by Laser light (PDT). The MTT assay was used to determine the cell viability.

### Clonogenic cell survival assay

Clonogenic cell survival assay was used for evaluation of the long-term proliferative potential of MDA-MB-231 cancer cells following PDT treatment. For this purpose, the treated cells were seeded at a density of 200 cells per well in 6-well plates and cultured for 7 days. Then, the media was removed from each plate and stained by 0.5% crystal violet in methanol^47^. The assay was done in triplicate.

### Determination of apoptosis by fluorescence microscopy and flow cytometry

MDA-MB-231 cells were treated with 0, 10, and 50 µg/mL ZrP-MB for 1 h and then illuminated (PDT). the cells were washed with PBS and then were dissociated by trypsinization, collected by centrifugation at room temperature, and washed twice with PBS. Morphological changes due to apoptosis induction by photodynamic therapy mediated ZrP-MB were detected by Acridine Orange/Ethidium Bromide (AO/EB) double staining using fluorescence microscopy (BEL, Italy) according to previous procedures^48–50^. All samples were stained and analyzed immediately at room temperature. For quantitative analysis of apoptosis induction by photodynamic treatment mediated ZrP-MB, the cells were collected as described above and then were suspended in 500 µL binding buffer containing 5 µL FITC Annexin-V and 5 µl PI reagents. Cells incubated for 15 min in dark condition and room temperature, and then were analyzed using flow cytometer (FACS Calibur, USA).

### ROS detection

Quantitation of ROS generated by MB and ZrP-MB in cells was performed by 2′,7′-dichloro dihydrofluorescin diacetate (DCFH-DA, Sigma-D6883). DCFH-DA passes easily through the cell membrane where it is deactylated to a non-fluorescent dihydrofluorescein, and an increase in fluorescence signal can be observed upon its oxidation. After PDT, the cells were trypsinized and, DCFH-DA was added to the medium at a final concentration of 2 mM. The plates were incubated for 45 minutes in the dark (37 °C, 5% CO_2_). Then medium containing DCFH-DA was removed and washed twice with PBS. The fluorescence emission of ROS can be detected by using excitation at 488 nm (blue) and emission at 530 nm (green) wavelengths. The generation of ROS was analyzed by Becton-Dickinson FACS Calibur Flow Cytometer (USA) using flowJo 7.6.1 software.

### Statistical analysis

All values are expressed as means ± SD. Results are expressed as with *n* denoting the number of experiments. P < 0.05 (*) was considered as statistically significant.

### Ethical approval

We didn’t perform any human or animal tests. So our work does not need any ethical approve for experiments.

## Results and Discussion

### Synthesis, intercalation and characterization of nanoparticles

**Figure 1:**
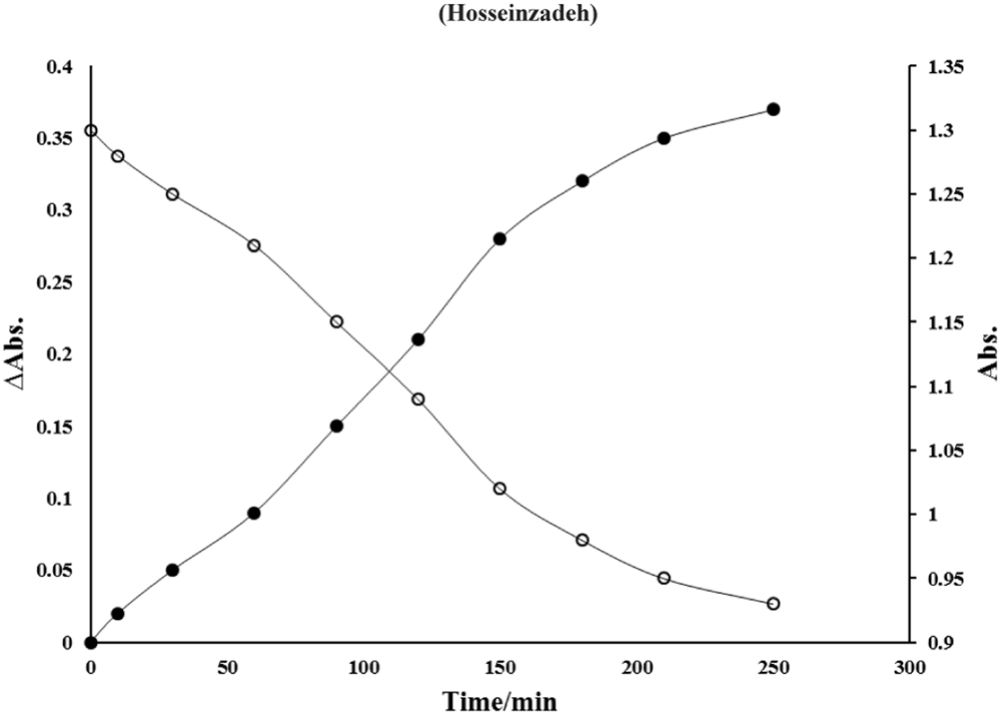
Alteration of methylene blue bulk Abs (○) and related ∆Abs (•) versus time (minute).

Synthesized nanoparticle was used for intercalating of methylene blue based on cation exchange potential of zirconium phosphate bilayer nanostructures. Since the methylene blue is a small cationic thiazine dye (methylthioninium chloride), in the presence of cation exchange material such as ZrP, the cationic dye exchange with interlayer H^+^. When the concentration of dye cation is high in solution, according to the thermodynamic rules, the chemical potential of dye in the bulk solution (and its related Gibbs free energy) is higher than intercalated dye. Due to the nature of spontaneous reactions; dye intercalated in the ZrP bilayers for decreasing Gibbs free energy of solution until the exchange equilibrium reach. Figure 1, represents the dye absorbance (λ_max_ = 664 nm) variations at different time intervals of ZrP-MB incubation at 25 °C and pH = 7.4.

**Figure 2:**
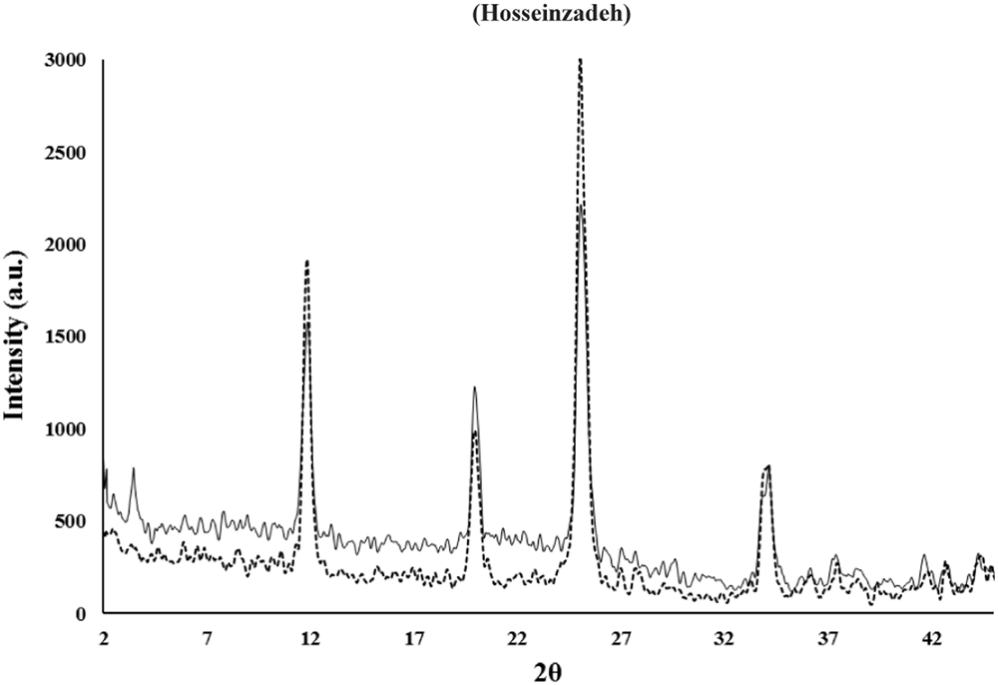
X-ray diffraction patterns of ZrP (−) and ZrP-MB (−) nanoparticles.

The absorbance was decreased by increasing incubation time until the equilibrium reaches. After equilibrium, the absorbance didn’t show any significant changes. The bare and intercalated nanoparticles were characterized using various techniques. Figure 2 shows the XRD pattern of synthesized nanoparticles.

According to the previous reports, appeared peaks in plans 002 at (2θ = 11–12 (/degree), 110 at (2θ = 19–20 (/degree) and 112 at (2θ = 24–26 (/degree) are the characteristics peaks in α-ZrP crystalline structure and no other impurity peaks were detected. (002) diffraction plane could be used for determination of the interlayer distances using the Bragg’s Law for diffraction pattern of zirconium phosphate, and the (001) diffraction plane is useful for approving and determination of the intercalation products and composite interlayer distance^33,51^. Based on the Bragg equation;

$$d=\frac{\lambda }{2\,sin\theta }$$

**Figure 3:**
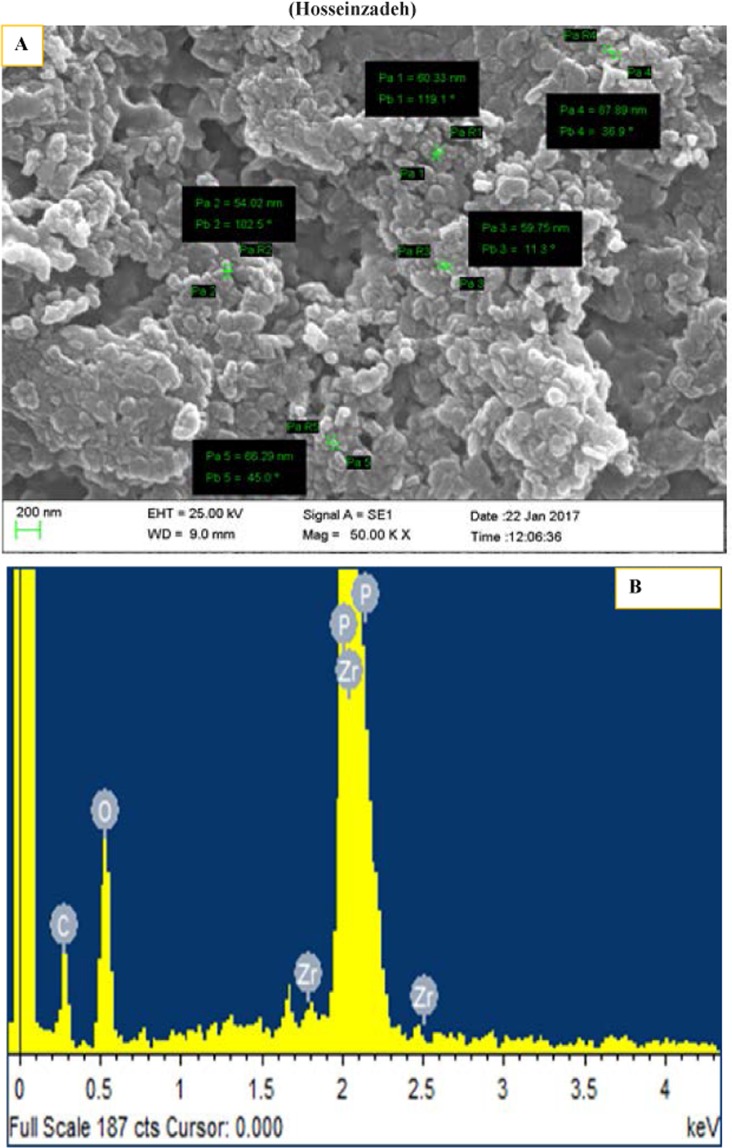
SEM image (**A**) and related EDS spectrum (**B**) of ZrP-MB nanoparticles.

The distance between planes (*d*) is equal to the ratio of the λ (wavelength of the source) and 2sin (θ; the diffraction angle). So, by using this equation, related parameters for synthesized materials can be determined. As mentioned above the 002 peak (2θ≈12) can be used for calculation of distance between layers of the ZrP (0.76 nm). Appearance of new peaks without any significant difference in the ZrP pattern approved the intercalation of methylene blue between interlayer of ZrP. As mentioned previously, the (001) diffraction plane was used for determination of the intercalation products and interlayer distance based on Bragg Law, (the obtained value is ≈14–17 Å). This expanding between ZrP crystalline sheets, demonstrated that methylene blue is injected into the interlayer distance. By considering the approximate dimensional parameters of methylene blue as 1.25–1.60 nm length, 0.57–0.84 nm width and thickness of about 0.5 nm, it seems that MB is intercalated longitudinally from top to bottom within the two sheet of ZrP by interaction of amine groups of MB with phosphate anions in the sheets (electrostatic interactions)^52^. According to the XRD pattern of intercalated into MB-ZrP, the pattern did not show any significant difference compared to pure ZrP nanocrystals indicating that ZrP remained unchanged during intercalation of methylene blue into the interlayer distance. The interlayer distance in α-ZrP crystalline structure is about 7.6 Å where 1 Å occupied with interstitial water molecules in the interlayer gallery^32^. A new diffraction peak (2θ≈4) is presented in the methylene blue intercalated zirconium phosphate that demonstrated the intercalation of methylene blue into the interlayer distance of ZrP^32^. The size and morphology of the synthesized nanostructures were determined using scanning electron microscopy (SEM). Also, energy-dispersive X-ray spectroscopy (EDS) analysis was carried out on a scanning electron microscope. SEM images and EDS graphs were shown in Fig. 3.

**Figure 4:**
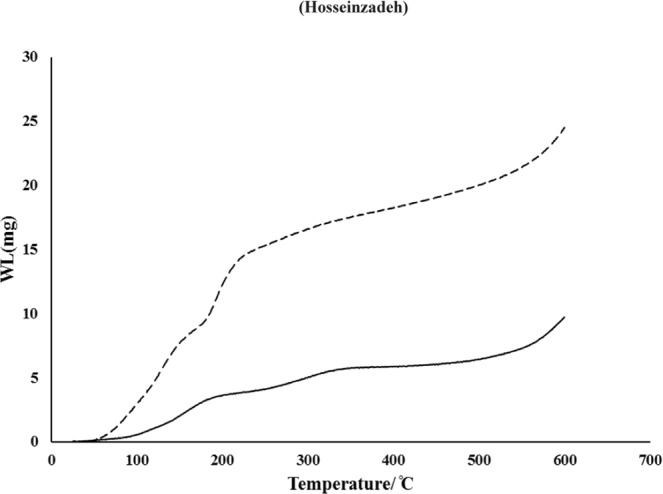
Thermogravimetric analysis (TGA) of ZrP (−) and the ZrP-MB (−).

The Fig. 3A shows uniform hexagonal morphology and layered structure with particle sizes ranging from 50 to 100 nm. Energy-dispersive X-ray spectroscopy analysis results (Fig. 3B), shows the Zr, O, P and C elements were incorporated into the system. The Zr, O, P and C elements homogeneously distributed throughout the prepared nanostructures. Figure 4 represents the thermal gravity (TG) graphs related to void ZrP and methylene blue intercalated ZrP.

Thermal gravity analysis (TGA) showed that methylene blue intercalated into the Zirconium phosphate nanolayeres and maximum 30–35% loading was achieved. 30% loading in synthesized nanoparticles considered in further experiments. These results are in agreement with obtained results from spectrophotometric and EDS measurements.

### Biocompatibility of ZrP nanoparticles and *in vitro* cytotoxicity of ZrP-MB

**Figure 5:**
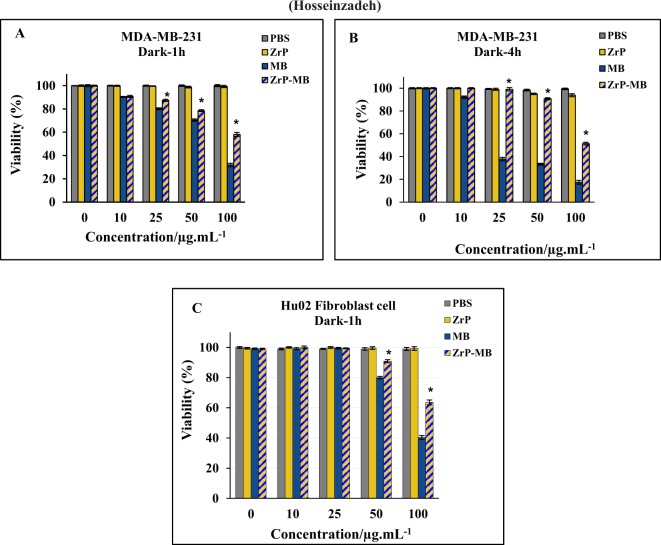
*In vitro* dark-toxicity of different concentrations of MB, based on MB contents, in ZrP-MB, ZrP, free MB and related blank (PBS) on MDA-MB-231 (**A**, 1 h incubation), MDA-MB-231 (**B**, 4 h incubation) and fibroblast HuO2 (C, 1 h incubation) cells, without laser irradiation. The results are expressed as mean ± SD (n = 3), *P < 0.05 (compared with MB treated group).

The effect of the zirconium phosphate nanomaterials on cell survival and cell proliferation was determined using MTT assay on MDA-MB-231 human breast cancer and human normal fibroblast cell lines. As reported previously, the incubation time can affect the dark toxicity of methylene blue, Santos *et al*., reported that the dark toxicity of methylene blue increased by increasing the incubation time (more than 1 hour) at low MB concentrations in MCF-10A, MCF-7 and MDA-MB-231 cells, but at higher concentration of MB, dark toxicity of MB on MCF-10A and MCF-7 cells increased at short incubation times, but has little effect on MDA-MB-231 cell viability in similar incubation times^53^. To obtain the dark cytotoxicity of methylene blue and hybrid nanomaterials, the MDA-MB-231 cells were treated with different concentrations of MB, ZrP-NPs and ZrP-MB ((concentration is shown in Fig. 5A,B) for 1 and 4 h incubation times, respectively. As it can be seen, the void ZrP nanoparticles did not change the cell viability upon changing the incubation time which shows the biocompatibility of ZrP nanoparticles. According to the results the MB dark toxicity is increased upon increasing the concentration of MB (more than 10 µg/mL) and incubation time more than 1 hour didn’t show any significant effect on cell viability. It is clear that the dark toxicity of MB is decreased by using ZrP as drug delivery system and treating cell with ZrP-MB decreased the dark toxicity compared to free MB in dark condition. For analyzing the effect of MB and ZrP-MB on normal cells, we used the human normal fibroblast. As it can be seen in Fig. 5C, the cell viability did not change significantly in low concentration of treating materials up to 25 µg/ml and by increasing the concentration (50 µg/ml and 100 µg/ml) the cell viability decreased. According to the results, the dark toxicity of MB on normal cells is decreased by using ZrP-MB instead of MB. It can be suggested that this nano-system can reduce the dark toxicity of methylene blue in comparison with free methylene blue. This effect lead to selective and localized PDT mediated MB in cancer and tumor sites by illuminating laser light and inducing controllable cell killing.

### Laser irradiation effect on cytotoxicity of hybrid nanomaterials (*In vitro* photodynamic assay)

**Figure 6:**
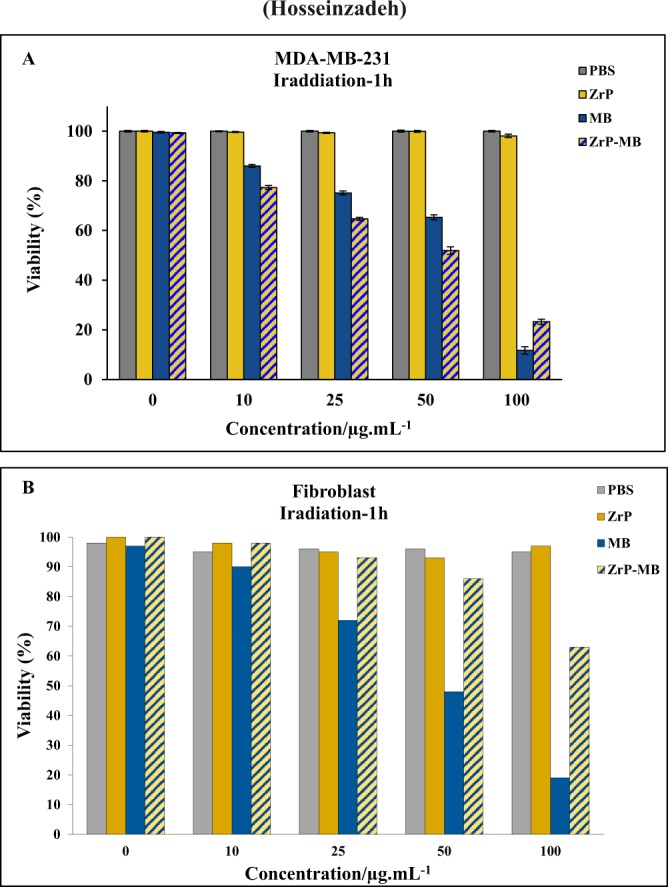
*In vitro* phototoxicity (laser irradiation) of different concentration of ZrP-MB, ZrP, MB and related blank (PBS) on MDA-MB-231 (**A**) and Fibroblast (**B**) cells after 1 h incubation. The results are expressed as mean ± SD (n = 3), *P < 0.05 (compared with MB treated group).

The effect of 660 nm laser light illumination (PDT) on ZrP-MB treated MDA-MB-231 cells (1 hour incubation) can be seen in Fig. 6A.

The results show that in the presence of laser irradiation the cell viability in control (only PBS) didn’t show any significant changes approve that the used dosage of light doesn’t show any photo-toxicity in the absence of photosensitizers. Also, ZrP treated cells didn’t show any significant changes in cell viability which approved the biocompatibility of synthesized layered ZrP nanostructures. The cell viability decreased by increasing photosensitizer (MB and ZrP-MB). In the presence of high concentrations of ZrP-MB and MB (100 µg/ml), cell viability decreased more in treated cells with MB than ZrP-MB. Interestingly, at lower concentrations of ZrP-MB and MB (lower than100 µg/ml) the cell viability decreased more in treated cell with ZrP-MB than MB. In other words, the results demonstrated that photodynamic efficacy of ZrP intercalated MB is higher than free MB (Fig. 5).

**Figure 7:**
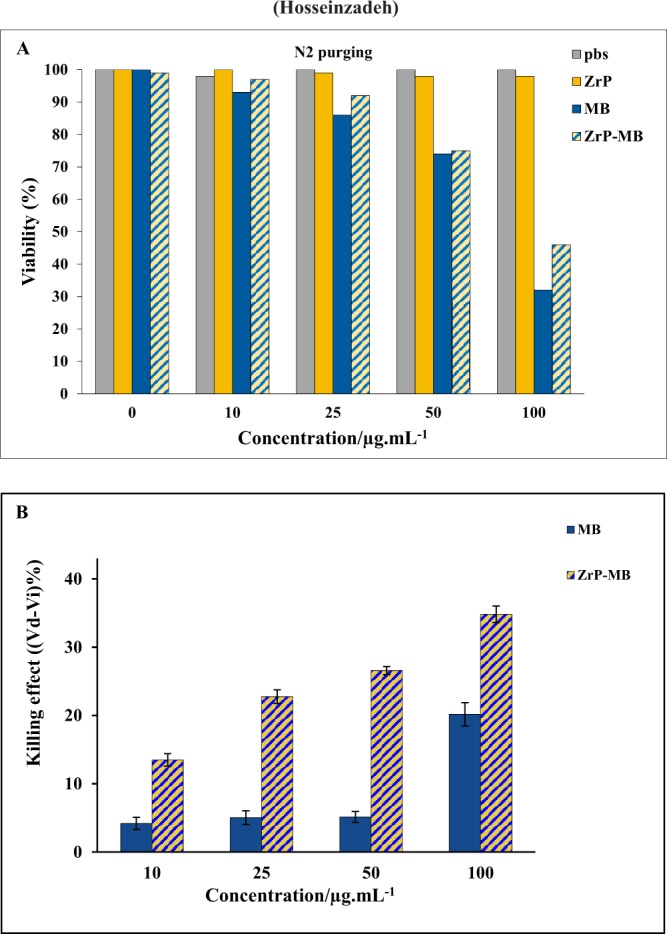
(**A**) Photodynamic activity of materials under N_2_ inert gas purging for examination of O_2_ effect. (**B**) Cell Viability (%) difference (V_Dark_ − V_Irradiation_) between dark and laser irradiation experiment for ZrP-MB, ZrP, MB treatments and related blank (PBS) on MDA-MB-231 cells, the results are expressed as mean ± SD (n = 3), *P < 0.05 (compared with MB treated group).

Figure 6B represents the effect of various concentrations of materials on the fibroblast normal cells under red light illumination. Due to the incubation time after treatment, the normal cells can repair the damage and can normalized the oxidative stress on the cells. As reported previously, due to the function of mitochondria in normal cells, the induced ROS materials can scavenged by antioxidant enzymes, such as superoxide dismutase, H_2_O_2_-removing enzymes, catalase, peroxidases, metal binding proteins, in the normal cells mitochondrial system due to the redox signaling activation^54–56^. However, in cancer cells dysfunction of mitochondrial system caused to increasing ROS materials in the cancer cells without scavenging of reactive oxygen spices, because of inactivation of redox signaling in the cancer cells due to mitochondrial dysfunction. So normal cells can repair themselves by activation of antioxidant ROS scavenging bio system but cancer cells don’t have this ability. Also it must be mentioned that the selective irradiation can decrease the damage to normal cells. By considering the cancerous parts as the targeting points for laser irradiation, the normal cell damages can be controlled and decreases^57–59^. Figure 7 depicted the molecular oxygen effect on PDT activity of treating materials (Fig. 7A) and photodynamic effect on cancer cells, after subtracting the dark toxicity for each treatment (Fig. 7B).

### Clonogenic cell survival assay

A Clonogenic cell survival assay was performed to evaluate the long term proliferative potential of MDA-MB-231 cells after PDT treatment. This assay used for determination of the ability of cells to proliferate indefinitely. The cells with this ability can form a large colony or a clone of cells which known as clonogenic cells. Various mechanisms are involved in cell death, however, the loss of cells ability in proliferation and propagation are known as the most common factor. So, the cells that remain the ability of proteins and DNA synthesis can go through one or two mitoses, but are unable to divide and produce a large number of population are known dead. The assay was considered for examination of treated cells with ZrP-MB nanostructures proliferation potential under laser illumination.

**Figure 8:**
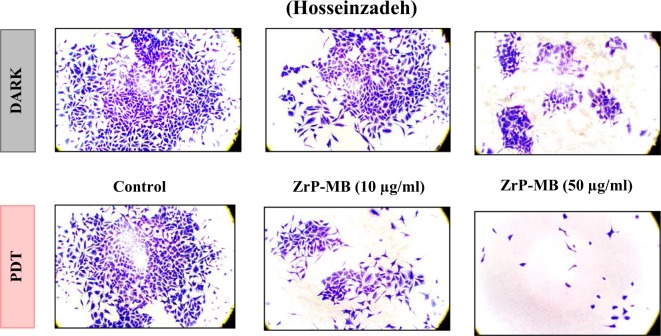
Clonogenic ability images (10X) of breast cancer cells at 0, 10 and 50 µg/mL of ZrP-MB at dark (control) and irradiation.

As can be seen in Fig. 8, the colony forming ability of the cells treated with various concentrations of ZrP-MB was decreased upon increasing the nanoparticle concentration in compare to control group (dark). It is clear that the PDT treatment by synthesized hybrid ZrP-MB nanoparticles induced cell death and decreased proliferation ability of MDA-MB-231 breast cancer cells.

### Apoptosis detection: AO/EB double staining and flow cytometry

To obtaining the apoptotic induction ability of ZrP-MB nanoparticles on MDA-MB-231 cancer cells, we treated the cells with (0, 10 and 50 µg/ml of ZrP-MB nanoparticle) and then PDT. The cells stained with Acridine Orange (AO) and Ethidium Bromide (EB) for fluorescence microscopy. Also the cells were stained with Annexin V-FITC and PI followed by flow cytometry analysis.

**Figure 9:**
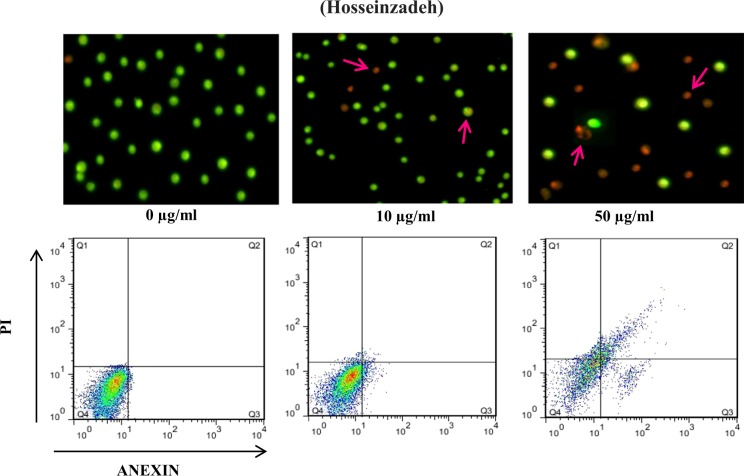
Apoptosis induction in MDA-MB-231 cells at 0, 10 and 50 µg/mL of ZrP-MB treatment under laser light irradiation using Acridine Orange (AO) and Ethidium Bromide (EB) double staining followed by fluorescence microscopy(40X) and annexin V-FITC and PI staining followed by flow cytometry. Arrows indicate apoptotic and necrotic cells.

As it can be seen in Fig. 9, under a fluorescence microscope, the normal cells (control) are green and have intact and round-shape nuclei. After treatment with ZrP-MB nanoparticle then PDT the cells turned to red (early or late apoptotic cells) with condensed or fragmental nuclei. Late apoptotic and necrotic cells were increased upon increasing ZrP-MB nanoparticle concentration (10 to 50 µg/mL). Annexin V binding and PI uptake is one of the most commonly method in measurement of cellular apoptosis and necrosis. The asymmetry of membrane phospholipids is perturbed in apoptotic cells and the loss of membrane conformational asymmetry causes the releasing of phosphatidylserine on the outer side of the plasma membrane. Annexin V binding to externalized phosphatidylserine can be used in measurement of cellular apoptosis. Also, apoptotic cells are characterized by DNA fragmentation and, consequently, that can be determined by staining with Propidium Iodide (PI) and following by flow cytometry assay. According to the obtained flow cytometry histograms (Fig. 9) related to ZrP-MB treatment at various concentrations and then PDT, it is clear that the apoptotic and necrotic cells were increased by increasing ZrP-MB concentration. As shown in Figs 5 and 6, the control shows 4% apoptotic cells (Q2 and Q3) whereas after treatment with 10 and 50 µg/ml of ZrP-MB then PDT, the early apoptotic cells represented 10%, 13% (Q3) and late apoptotic cells represented 5% and 30% (Q2) of the total cells, respectively. At 50 µg/ml of ZrP-MB 7% necrosis is also observed (Q1). It can be concluded that by increasing nanoparticles concentration the apoptotic cells increased.

### ROS generation after PDT with ZrP-MB

**Figure 10:**
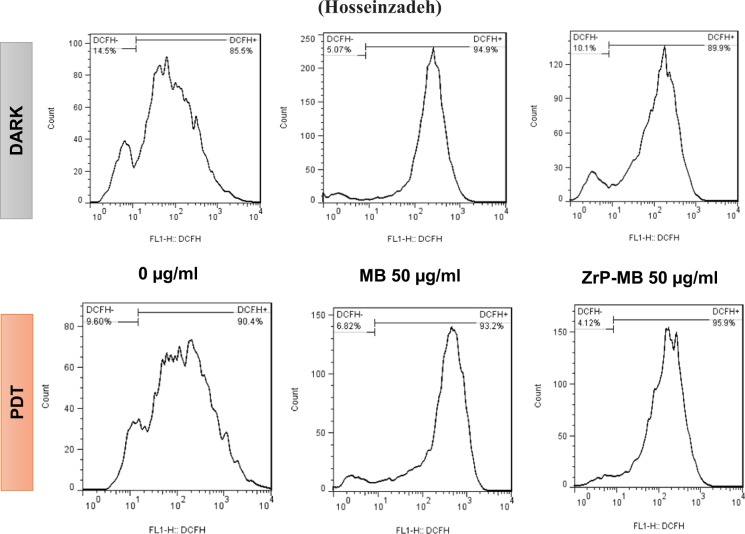
Effects of MB (50 µg/ml) and ZrP-MB (50 µg/ml) following PDT on intracellular ROS generation in MDA-MB-231 cells. The cells were stained with DCFH-DA (2 mM), followed by flow cytometry. DCFH-DA will be converted to DCFH^+^ in presence of reactive oxygen spices (ROS) and induce fluorescence signal in histogram.

In PDT, the excited photosensitizer transfers energy to molecular oxygen and generates cytotoxic reactive oxygen spices (ROS) which could induce oxidative damages or cell death^60^. However, it is not clear whether ROS generation is involved in cell death mechanism of ZrP-MB -PDT. To clarify it, we examined intracellular ROS generation in terms of flow cytometry by DCFH-DA. As it can be seen in Fig. 10, at dark condition the ROS generation in cells, treated with MB, is higher than the cells treated with ZrP-MB. While after PDT the cells that treated with ZrP-MB displayed higher ROS generation compared to MB treated cells.

According to the obtained results and considering various scientific research reports, it can be concluded that the synthesized nano-hybrid materials can be used for selective photodynamic treatment of cancer cells, in the presence of normal cells, due to the selective release of methylene blue because of cation exchange ability of zirconium phosphate in the acidic microenvironment of cancer cells^32–34,61^. By exchanging methylene blue cations with hydrogen ions (H^+^), the releasing of methylene blue was occurred and photo-activation of MB using red light LED illumination caused photodynamic cell killing on cancer cells, consequently^29,62^.

## Conclusion

In conclusion, we have synthesized and characterized ZrP NPs, and then methylene blue was loaded in the interlayer part of the nanoplatelets. The dark cytotoxicity experiments demonstrated that the synthesized nanoplatelets were biocompatible and didn’t show any significant cytotoxicity. The drug loading fine efficacy, swelling and water dispersibility were the characteristics of obtained nanoparticles. ZrP-MB NPs were effective in delivery of the methylene blue to MDA-MB-231 breast cancer cells. Dark experiments clarifyed that methylene blue cellular toxicity decreaseed by intercalating into the layers of ZrP nanoparticles. Photodynamic therapy using synthesized nanoparticles demonstrated that the intercalation can enhance the PDT efficacy based on decreasing methylene blue dark toxicity and prevention of methylene blue environmental redox reactions. In the other words, it could be stated that the methylene blue intercalation in ZrP nanoparticles increased selectivity in local photodynamic toxicity based on selective laser illumination. According to the acidic extracellular microenvironment of cancer cells and considering the characteristics of ZrP nanoparticles cation exchange ability, selective release of methylene blue in the cancer cells microenvironment can enhance the photodynamic therapy efficacy. The clonogenic assay, florescence microscopic image and flow cytometry experiments of treated cells confirmed the obtained results. The lack of cytotoxicity of zirconium phosphate nanoplatelets offered promising potential in application of ZrP nanoparticles as cationic photosensitizer’s carrier for effective photodynamic therapy. At the end, *in vivo* studies needed to evaluate the toxicity, feasibility and efficacy of ZrP-MB nanoparticles in breast cancer animal models.

## Data Availability

The datasets generated and analyzed during the current study are available from the corresponding authors on reasonable request by permission of institute and department chairman’s.
